# Sustained low-efficiency dialysis—an old method but possibly a new solution for environmental nephrology

**DOI:** 10.1080/0886022X.2025.2580065

**Published:** 2025-11-11

**Authors:** Aleksandra Rymarz, Filip Wantoch-Rekowski, Paweł Żebrowski, Zuzanna Jakubowska, Małgorzata Kępska-Dzilińska, Ewa Wojtaszek, Jolanta Małyszko

**Affiliations:** Department of Nephrology, Dialysis and Internal Medicine, Medical University of Warsaw, Warsaw, Poland

**Keywords:** SLED, CRRT, acute kidney injury, green nephrology

## Abstract

Slow low-efficiency dialysis (SLED) is viewed historically a ‘hybrid’ technique of kidney replacement therapy, representing a transition between intermittent and continuous kidney replacement therapy. It can be performed with a mobile single-pass batch dialysis system or multifunctional hemodialysis machines, using reduced dialysate flow and an extended procedure duration. We summarized the technical aspects, as well as the practical advantages of SLED. Moreover, we present our experience of using SLED in selected difficult clinical conditions, such as intraoperative renal replacement therapy during liver transplantation, in sepsis, and in heart failure patients with recalcitrant volume overload. In this paper, we aimed to describe the unique advantages of SLED in terms of efficacy, safety, and, more importantly, sustainability in kidney care in the intensive care units.

## Graphical Abstract

**Figure:**
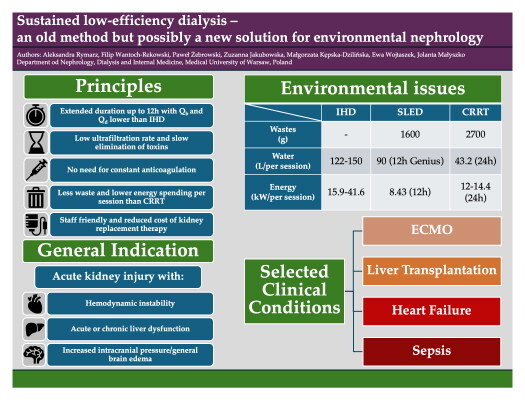

## Introduction

Over the last three decades, the incidence of acute kidney injury (AKI) has increased significantly with commensurate burden of kidney care needed [1,2]. Potential explanations for this trend include a greater sensitivity of AKI diagnosis associated with the consensus definition of AKI and better reporting, nonetheless, a ‘real’ growth of AKI prevalence is also likely to be present. Aging of the population and an increasing number of comorbidities, such as diabetes, obesity, hypertension, malignancy, and well-defined risk factors of AKI, such as sepsis, major surgery, and heart failure, are the reasons for this situation. The significant rise in the incidence of AKI has led to an increased number of patients requiring kidney replacement therapy (KRT). According to a multicenter study published in 2005, approximately 5% of patients treated in intensive care units (ICUs) received KRT, and the mortality rate in this group reached 50% [3]. In a multicenter, multinational analysis published ten years later, KRT was used in 13.5% of patients admitted to the ICU [4]. In a single-center analysis by Matuszkiewicz-Rowińska et al. the number of patients requiring KRT because of AKI quadrupled between 2004 and 2009 [5]. Given these data, providing the proper treatment of AKI requires engagement of numerous medical staff and significantly increases the costs.

Furthermore, the impact of ICU-based dialysis on the environment is unfavorable due to the high consumption of water, energy, and disposable plastic materials [6]. Therefore, the concept sustainability in kidney care is also gaining momentum during treatment of AKI. The first step of this strategy is prevention, early detection, and expeditious treatment of AKI to avoid the need for KRT. When this strategy is ineffective, the optimization of KRT procedures should be introduced [7].

Among hemodialytic modalities used in the treatment of AKI, key options include intermittent hemodialysis (IHD), continuous kidney replacement therapy (CKRT), prolonged intermittent kidney replacement therapy (PIKRT), and sustained low-efficiency dialysis (SLED). In some centers acute peritoneal dialyses-PD are also performed in AKI.

Continuous kidney replacement therapy (CKRT) was introduced in 1977 to improve homeostasis among hemodynamically unstable patients with resistance to diuretics [8]. It has been suggested that this modality provides superior hemodynamic and cardiovascular stability owing to hypothermia, which in turn increases venous return and blood pressure relative to intermittent hemodialysis [9–11]. Current practice of pump-driven continuous veno-venous hemofiltration provides higher global effluent rates and reasonable predictable net ultrafiltration to address multiple aspect of critical illness with AKI in ICUs. Due to cost issues related to CKRT, such as the need for sterilized solution bags for substitution fluids or dialysates, the concept and practice of extended daily dialysis concept was developed [12–14].

This review aims to critically evaluate the clinical, technical, and environmental advantages of SLED as a sustainable alternative to CKRT and providing benefits over intermittent hemodialysis (IHD) in critical care settings.

### Slow low efficient dialysis (SLED)

#### Principles and indications in general

Sustained low-efficiency dialysis (SLED) is a hybrid kidney replacement therapy (KRT) that uses lower blood flow (Qb) and lower dialysis fluid flow (Qd) than those used in intermittent dialysis. In this aspect, as an amalgamation of IHD and CKRT, average Qb is 100–300 mL/min, and Qd is 100–350 mL/min.

In the SLED method, the time required to achieve an adequate clearance of the molecules is 6–12 h, which is an extended duration compared to classic hemodialysis, and much lower blood flows like CKRT. It allows for maintaining the advantages and reducing the disadvantages of both methods. However, some centers use up till 22–24 h-day sessions of SLED, whereas Qb is sufficient at rate of 50–100 mL/min, yet retaining excellent clearance. This prolonged procedure time allows for a relatively low ultrafiltration rate per hour, which is associated with maintaining the patient’s hemodynamic stability. SLED, a classic dialysis procedure based on the diffusion process and utilizing filters typical for this method, can be performed without anticoagulation using intermittent saline flushes, which is particularly beneficial in patients with bleeding risk. This is a significant advantage, enabling the use of this procedure in patients with contraindication to heparin-based anticoagulation as well as to anticoagulation based on citrates. CKRT is sometimes performed without anticoagulation. In one of the randomized controlled trials, the mean filter lifespan was 14.3 h for CKRT without anticoagulation, which makes the duration similar to SLED [15]. However, during this time, the dialysate rate is significantly lower than in the SLED procedure. It is also worth noting that to perform CKRT without anticoagulation, a higher blood flow is usually required, which can lead to hemodynamic instability.

SLED, historically called a ‘hybrid’ method, offers CRRT-like advantages such as a low ultrafiltration rate, which provides hemodynamic stability and allows for flexible adjustment to fluid balance, and a slow removal of uremic toxins, thereby avoiding significant fluctuations and fluid shifts [16].

These features are associated with the main indications for using SLED in the treatment of AKI. The crucial indication is hemodynamic instability, which is often present in patients treated in intensive care units (ICU), cardiac intensive care units, or undergoing surgery [17]. A slow toxin elimination rate, which reduces the risk of disequilibrium syndrome, is an indication for SLED, which is particularly important for patients with significant electrolyte disorders and those with present or impending cerebral edema, such as those with various forms of craniocerebral trauma. The ability of adjusting dialysate sodium concentration in certain platform is an additional advantage of SLED technology. The possibility of performing KRT without anticoagulation is essential in patients just before, during, and after surgery or with coagulation disorders. Acute or chronic liver dysfunction is an example when heparin usage is problematic, and citrate administration is contraindicated. Therefore, SLED performed only with saline flushes is an ideal solution in this situation.

KDIGO guidelines for AKI suggest introducing IHD and CKRT as complementary methods and recommending switching between them. CKRT is suggested in hemodynamically unstable patients as well as in patients with acute brain injury or other causes of increased intracranial pressure or generalized brain edema [18].

As a hybrid method with the advantages of CKRT, SLED can be used for the same indications as CKRT. Clinical trials did not prove the superiority of CKRT over SLED [19]. Therefore, it seems reasonable to introduce SLED when the indications above exist and before CKRT is considered. As SLED lasts 6–12 h, this method can easily be switched to CKRT. This therapeutic sequence, where SLED is the first choice and CKRT is the next step in case of insufficient metabolic efficacy of SLED, allows for reducing the cost of KRT and is staff friendly.

However, special machines are also dedicated to this kind of treatment. One example is the Genius system, which enables the production of dialysis fluid on demand, just before the dialysis session. Dr. B. Torstegeen devised this system in the 1970s and later refined it over the following decades [20]. In this review, we will also share our experiences of using the hospital’s system, which we have been using for over 20 years. However, the production of this type ended in 2024 and this system is no longer available.

While systems like the Genius offer an integrated batch solution, SLED therapy can be successfully performed using standard, modern hemodialysis machines commonly found in most intensive care units (ICUs) and dialysis centers. The key is to adjust the treatment parameters—primarily by lowering the blood (Qb) and dialysate (Qd) flow rates and extending the duration of therapy. This approach does not require specialized equipment dedicated solely to SLED, making the technique widely accessible and flexible to implement.

Slowing the dialysis flow rate to 100–200 mL/min and the ultrafiltration rate while maintaining a more extended dialysis permits the treatment of patients with hemodynamic instability, sometimes even conducted continuously at minimal flow rates, ensuring that all changes occur gradually [21]. This allows for the safe removal of fluid and toxins, even in critically ill patients in the ICU [22].

In contrast to CKRT, SLED can be performed without heparin with conventional equipment, just like the equipment used in IHD, and is typically conducted overnight, allowing for many medical procedures, such as rehabilitation or diagnostic tests, to be performed during the day. A comparison of different extracorporeal techniques is presented in Table 1.

**Table 1.** Comparison of different methods of extracorporeal techniques.

	IHD	SLED	CKRT
Duration	4h 3 times per week	Varies from 6 to 12 h daily	24 h per day
Blood flow	300–400 ml per minute	100–200 ml per minute	100–200 ml per minute
Hemodynamic stability	Low with high-risk hypotension in critically ill patients	Superior	Superior
Clearence	Mostly low molecular weight toxins	Low and medium molecular	Low and medium molecular
Anticoagulation	Can be done without	Can be done without	Constant, but it can be also done without anticoagulation
Dialysate flow	Up to 500 ml/per minute	100–200 ml per minute	30–60 ml per minute
Tolerance of ultrafiltration	Sometimes problematic	Good	Good
Urea clearance per minute	High	Moderate	Low
Cost	High	High	Very high
		Low with Genius single-pass batch system	
Wastes	–	1600 g [26]	2700 g (+substitution volume up to 15000 g) [26]
Water	120–155 L/per session [26]	144 L/per 12h session (self-calculation)	43.2 L/24 h [27]
		90 L/per 12 h Genius	
Energy	5.3–10.4 kWh [27]	5.71 kW/per 8h session	12–14.4 kW/24 h [27]
	15.9–41.6/per session	8.43 kW/per 12h session [27]	

The Genius system utilizes a single-pass batch for hemodialysis, including a closed reservoir and dialysate circuit with a high-mobility machine. Before each session, the batch is filled with pure, bacteriologically safe dialysis fluid under positive pressure with an automated process. In such batch systems, a 75–90 liter volume of dialysate is prepared before therapy commences within the machine’s tank. This process involves mixing sterile concentrate with purified water from a reverse osmosis (RO) system (either stationary or portable). The prepared, heated, and buffered dialysate is contained in a closed circuit, ensuring its sterility throughout the treatment. When using standard dialysis machines, dialysate is generated continuously (online) from a central water treatment system or from individual, portable RO units at the patient’s bedside. Nowadays In USA there are two systems approved by FDA i.e. Tablo® Hemodialysis System registered perform all the following modalities: HD, SLED, PIRRT and isolated ultrafiltration and QuantaTM Dialysis System registered to perform HD, SLED and CRRT.

When dialysis is required in a different hospital unit, the mobile system is easily transported and managed by the personnel overseeing the patient’s care.

In the Table 1 comparison of different methods of extracorporeal techniques is provided.

#### SLED as a green modality

Climate change is occurring at such a rapid pace that sustainable development is considered one of the significant global challenges of our time. Dwindling water resources and widespread plastic pollution pose additional threats. Unfortunately, debates about the scope and nature of actions required to combat climate change remain contentious and complex due to competing political and economic priorities [23]. Initiating dialysis therapy involves significant costs in terms of waste, water, and energy consumption, as well as the need for a specialized team.

Conventional hemodialysis (HD) with high dialysate flow rates is one of the most demanding treatments in terms of water, energy, and consumables; yet, its negative environmental impact is still largely overlooked [24]. Going forward, efforts must be made to manage water consumption during dialysis sessions while providing adequate and effective dialysis, with consideration for the patients’ needs [25]. Slow, low-throughput daily dialysis in the context of sustainable nephrology may offer some ways to minimize the environmental impact. Nursing also represents a resource with significant cost (human labour). Developing the SLED program, certainly in the high-income countries, i.e., the US - enables the integration of the workflow of dialysis into the workflow of nurses in the ICUs; thus, alleviating the need for dialysis nurses to be at bedside and with preparations around a 5 h burden and turning it into 1 single nurse being able to provide all the needs of a critically ill patients—including the daily need for KRT.

Precise data regarding this impact is very limited. The expenditure of water and energy, as well as waste generation, depends mainly on the type and brand of renal replacement therapy equipment. Traditional dialysis methods require significant amounts of water. In a conventional 4-h dialysis session, the water used at a fluid flow of 500 mL/min (standard dialysis fluid flow) is 120 liters [26]. In general, this one is not even accounting for the ‘reject’ water during reverse osmosis generation. Hence, the actual water usage is much large. In a 12-h SLED procedure performed using the mobile Genius Fresenius system, 90 liters or 74 liters of fluid are used, which corresponds to the amount of fluid in a single batch. However, different SLED devices use significantly more water. Sala et al. report the water consumption in SLED as 237 L per 8-h session or 345 L per 12-h session using Fresenius Medical Care 5008 machine but for dialysate flow rate 300 mL/min [27]. In the same analysis, water consumption for CKRT using the Baxter Prismaflex was calculated to be 43.2 L per 24 h for an 80 kg patient, with a dialysate flow rate of 1300 mL/h and a reposition solution flow rate of 500 mL/h. On the other hand, waste generation and energy consumption were much higher for CKRT (Table 1) [26,27]. This paper was published only in the abstract form [27]. Considering the advantages of low energy consumption and low waste generation for SLED techniques, higher water consumption can be offset by the reutilization of rejected water in non-medical applications, such as agriculture or horticulture. This argument would make sense only for in-center dialysis units (i.e., recycling the purified, but ‘reject’ water by a central reverse osmosis), which represent the only HD modality in many countries.

In the context of these data, the system, which utilizes a disposable dialysate tank, eliminates the need for continuous solution preparation, thereby reducing the workload of the pump and heater, and consequently, consumes less energy per treatment, making it seem environmentally friendly. However, the solutions for sustainability in nephrology, must be balanced against the primary goal of providing optimal clinical care. Resource conservation, such as reducing water consumption, cannot come at the expense of treatment efficacy. An analogous debate has been observed in chronic kidney disease care, where hemodiafiltration (HDF), which consumes more water than standard hemodialysis (HD) due to the need for substitution fluid, has proven its superiority in reducing cardiovascular and all-cause mortality [28]. Therefore, when selecting a KRT modality for AKI, the environmental benefits must be weighed against the potential impact on clinical outcomes, such as the adequacy of uremic toxin removal.

Moreover, although the Genius system employs the same standard chemical disinfection protocols and reverse-osmosis water treatment, its batch-wise dialysate preparation drastically reduces the reverse osmosis system’s active runtime, leaving the unit mostly in standby [29].

SLED, as a hybrid technique, should be considered to optimize the treatment process. Technologies that effectively combine intermittent dialysis and continuous renal replacement therapy can shorten treatment time and, consequently, reduce the number of resources needed, which is beneficial for both patients and the environment.

In the first observational, prospective pilot study on SLED in the ICU, Berbece and Richardson [12] compared cost, anticoagulation, and small solute removal by SLED and CKRT. For CKRT, either heparin or citrate is used, while for SLED, either saline or saline flushes are used. They found that the weekly cost for SLED was $1,431, whereas the costs for CKRT with heparin were $2,607 and $3,089 for citrate anticoagulation. They emphasized that the cost of filter sets and dialysate represented a significant cost difference between SLED and CKRT. In addition, Kt/V for SLED was significantly higher compared to CKRT, with similar solute removal equivalent to CKRT, but at a lower cost. In their study, SLED was performed using conventional HD machines (Integra, Gambro Canada, Richmond Hill, Ontario, Canada). They also stated in the discussion section that most of the SLED descriptions originated from only three centers, which used Fresenius dialysis machines [30–32]. The renal replacement therapy study in ICU patients (RESCUE) is a prospective, randomized intention-to-treat study, conducted to compare two different RRTs in critically ill patients with AKI, was performed between 1 April 2006 and 31 January 2009 in the surgical ICU of the University of Heidelberg, Germany. Schwenger et al. [33] assessed the cost of SLED-BD treatment (12-h of dialysis with a blood flow rate of 100 to 120 mL/min) or CVVH. For all SLED treatments, high flux polysulfone filters were used. If conventional high-flux membranes were used instead of specific AKI membranes, the cost difference increased to €1,300 per patient favoring SLED, and this modality was associated with significantly lower costs even after consideration of the higher reimbursement for CVVH by the German DRG system. As stressed by Marshall et al. [34] SLED can be performed without the need for systemic anticoagulation with reduced costs as compared to CKRT. In addition, nocturnal SLED, where available, allows patients to be transported outside the ICU during the daytime hours for routine tests and procedures, without concern about interrupting the KRT session. This is mostly an issue whether the SLED workflow is properly integrated into the workflow of ICU nurses.

A meta-analysis by Dalbhi et al. [35] demonstrated that the cost-effectiveness of SLED is superior to that of CRRT. Other advantages of performing SLED instead of CKRT include easy availability and a reduced need for specialist expertise, as well as a lower requirement for anticoagulation compared to CKRT, with a comparable ability to achieve adequate renal replacement in hemodynamically unstable patients. In addition, this meta-analysis also showed that there were no significant differences in the length of ICU stay or the rate of fluid removal between patients receiving SLED and those receiving CKRT [35] Promoting such innovations in nephrology can support not only patient health but also environmental protection, which is crucial in the context of climate change and the need for sustainable development.

#### Efficacy of SLED

AKI in the ICU is a common complication associated with increased mortality [36]. In a multicenter analysis based on 1800 patients from 97 centers, AKI developed during the first week of hospitalization in the ICU in 57% of patients, and 13.5% of them required KRT [4].

There are three types of extracorporeal kidney replacement therapies available in the ICU: intermittent hemodialysis (IHD), continuous kidney replacement therapy (CKRT), and prolonged intermittent kidney replacement therapy (PIKRT), including sustained low-efficiency dialysis (SLED). Technical differences between these methods are described in Section 2. Decision-making regarding the modality of KRT for a patient treated in the ICU is based mainly on the patient’s hemodynamic status. In case of hemodynamic instability, CKRT or SLED is recommended [11]. Among the advantages of SLED in comparison to CKRT, the following factors are enumerated: the procedure lasting 6–12 h can be performed during the night hours, and other therapeutic or diagnostic procedures available only during the day can also be performed, lower costs of SLED than CKRT and a decreased workload for the ICU nursing staff. Considering these advantages of SLED, the question of effectiveness arises.

Contemporary ICU practice often favors continuous renal replacement therapy (CRRT), which is perceived as straightforward to implement by intensive care staff, frequently without continuous, direct nephrology oversight. However, the predominantly convective transport in CRRT, although effective for middle-molecule clearance, may be less efficient in eliminating small, water-soluble toxins compared to the diffusive mechanism that underpins SLED. The extended duration of SLED, combined with efficient diffusion, enables effective small-solute clearance, which is crucial for controlling azotemia and electrolyte disturbances.

In the German study from Heidelberg, mortality rate and hemodynamic stability did not differ between SLED and CKRT. However, SLED treatment was associated with a lower quantity of blood transfusions, shorter duration of mechanical ventilation and ICU stay, faster recovery of renal function, less time spent on this procedure by the staff, and lower costs for providing KRT [33]. Similar data were reported by Baldwin et al. [37].

A meta-analysis by Zhan et al. based on RCTs, did not show a difference in mortality rates in patients treated with extended daily dialysis (EDD), defined by a duration of 6–24 h, compared to those treated with CKRT. However, in observational studies, patients treated with EDD had a lower risk of death than patients treated with CKRT (relative risk, 0.86; 95% CI, 0.74–1.00; *p* = 0.05). There was also no significant difference in the recovery of kidney function, fluid removal, or the duration of stay in the intensive care unit. EDD showed similar biochemical efficacy to CKRT during treatment. Serum urea, creatinine, and phosphate were not significantly different in these two modalities [38]. Lonnemann et al. [39] also stressed that extended high-flux dialysis combines the benefits of CKRT (good cardiovascular stability, sterile dialysate) with the advantages of intermittent dialysis (high urea clearance, low treatment costs).

A published meta-analysis of a total of 1564 patients from 18 studies by Kovasc et al. [40] primarily compared the mortality rate, renal function recovery, and length of stay in the ICU. There were no differences in primary outcome, overall proportion of renal recovery between SLED and CKRT as well as in the secondary outcome of time to renal recovery. However, statistically, SLED was marginally favored over CKRT for the mortality, the secondary outcomes.

Nash et al. [41], in a meta-analysis compared the efficacy of CKRT, SLED, and IHD. They did not observe any significant differences between CKRT and SLED in terms of ICU, in-hospital, or 30-day mortality rate, dialysis dependence, or length of stay [41]. In a meta-analysis by Zhao et al. no significant differences were observed in renal recovery and in-hospital mortality between patients treated with CKRT and SLED [42].

The overwhelming majority of analyses showed no significant differences between CKRT and SLED. However, in a study performed by Sun et al. [43], the authors observed greater improvement of kidney function in patients with sepsis and septic shock treated with continuous veno-venous hemofiltration (CVVHF) compared to those treated with extended daily hemofiltration (EDHF) (50.77% in the CVVHF group versus 32.50% in the EDHF group, *p* = 0.026). The 60-day mortality did not differ between the groups [43]. A longer duration of treatment was more effective in this specific type of illness, associated with a high catabolic rate and a significant inflammatory reaction. AKI is a heterogeneous syndrome, not a single disease, and different pathophysiological mechanisms lead to its development. Therefore, current studies on AKI try to extract different sub-phenotypes and endotypes of this syndrome, which could lead to personalized and more precise treatment of this condition [44]. One of the sub-phenotypes of AKI that has been extracted is sepsis.

Given these data, our opinion is that SLED can replace CKRT in most cases or can serve as a bridge between IHD and CKRT.

Despite these obvious advantages, SLED remains underutilized in ICUs. According to a survey performed among specialists working in ICUs, mainly in Europe, 90% of them use CRRT, 60% use IHD, while only 24% use SLED for AKI treatment [45].

As we performed retrospective analysis based on the data obtained from medical records of 774 patients with AKI, as defined by KDIGO criteria, requiring dialysis (AKI-D) in our single university hospital between Jan 2005 and Dec 2009, we observed that the incidence of AKI-D increased from 52 to 202 cases per million person-years, reaching 241 per million person-years in 2009. At that time, we introduced new types of dialysis machines—Multifiltrate and Genius 90—which enabled the performance of continuous and hybrid techniques on-site. To this day, in some cases, we are still asked to perform SLED instead of CKRT in our ICU [5].

### SLED in selected clinical conditions

#### SLED in liver transplantation

Orthotopic LT is a lengthy, technically complex procedure with serious hemodynamic and metabolic challenges. The hepatectomy phase is associated with a significant risk of massive blood loss, particularly in cases involving expanded collateral circulation and coagulopathy. During surgery, the anhepatic phase is characterized by progressive metabolic acidosis (absence of citrate/lactate metabolism) and worsening coagulopathy (lack of clotting factor production). Concurrent kidney dysfunction would further aggravate the metabolic and hemodynamic alterations described above. Thus, patients with renal failure are considered to run an exceptional risk of metabolic acidosis and hyperkalemia. During graft reperfusion, a crucial phase in the procedure, a surge of acidotic, cold, and hyperkalemic blood is released into the recipient’s circulation, which may result in significant hemodynamic instability, known as postreperfusion syndrome (PRS). Acute kidney injury is a common dysfunction in patients with progressive liver failure who are qualified for liver transplantation (LTx). The estimated incidence of AKI among liver recipients before operation is 10–30% [46]. Renal replacement therapy is required in 10–20% [47].

Because of liver failure, some pathophysiological mechanisms develop, leading to kidney dysfunction and can complicate renal supportive treatment. Hemodynamic instability, acidosis with high levels of lactate, and hyponatremia are often observed in liver transplant recipients waiting for LTx. These disturbances indicate continuous or prolonged KRT rather than short, intermittent dialysis. Most of the patients requiring KRT before LTx also need this therapy during the transplantation. The procedure of LTx is associated with the risk of electrolyte, acid-base, and fluid disturbances, which make intraoperative management extremely difficult, especially in patients with kidney dysfunction. One of the complications appearing during the operation is hyperkalemia. It is associated with postreperfusion syndrome and/or with a high amount of packed red blood cell (PRBC) transfusions. Hyponatremia, preexisting before liver transplantation (LTx), requires slow equilibration during the operation to avoid myelinolysis. Acidosis resulting from kidney and liver failure requires slow, gradual correction [48]. The LTx procedure is associated with significant fluid infusions and PRBC transfusions, leading to fluid overload with the increased risk of pulmonary or brain edema. These factors suggest the need to apply KRT and favor continuous or prolonged KRT for this intraoperative procedure.

Another problem with intraoperative KRT during LTx is anticoagulation. Heparin-based anticoagulation is not ideal due to the operation itself, and disturbances associated with liver failure, such as the decreased production of coagulation factors (I, II, VII, IX, and X), thrombocytopenia, and others. Regional anticoagulation with citrates brings a risk of their accumulation and exacerbation of acidosis in patients with liver dysfunction. KRT conducted only with a saline flush is a solution that SLED can provide. There are several studies on the safety of citrate anticoagulation in liver failure [49,50], as well as in patients receiving plasma adsorption plus plasma exchange therapy [51], also including systematic review and meta-analysis by Zhang et al. [52]. Pourcine et al. [53] investigated retrospectively the safety of regional citrate anticoagulation-RCA in patients with liver impairment during SLED, performed using conventional HD machine. They did not observe citrate accumulation but recommended acid-base status monitoring together with electrolytes to ensure safety. Lahmer et al. [54] in the prospective study assessed safety and feasible of SLED (performed using single-pass batch dialysis system) in patients with liver failure using a citrate anticoagulation. They also stressed that despite substantial accumulation of citrate in serum, SLED is both safe and feasible in these patients; however, they also recommended careful monitoring of electrolytes and acid-base status to ensure patient safety. Moreover, Szamosfalvi et al. [55] designed a protocol for sustained low-efficiency dialysis using regional citrate anticoagulation for critically ill patients. Clinically, this SLED-RCA protocol in an anhepatic, *ex vivo* dialysis system, completely abrogated clotting, without adverse electrolyte effects.

The only one study by Pertica et al. [56] described use of citrate in 5 patients requiring CKRT after liver transplantation. The KDIGO guidelines do not recommend anticoagulation based on citrates in patients with severe liver failure therefore they should not be treated as a standard procedure in this group of patients [18].

The frequency of intraoperative KRT (IOKRT) during liver transplantations is 10–40% [57,58]. In our academic center, 14.5% of liver recipients required IOKRT in 2024 (personal information, abstract accepted for ASN 2025, nr 4347220). In a single-center study from the US, Nadim et al. [59] observed the necessity of IOKRT in 32% of liver transplant recipients. Available data about the methods used in IOKRT are scarce. SLED was a method of IOKRT in the study performed by Koscielska et al. [57]. The analysis included 102 liver transplant recipients who underwent SLED procedure during LTx with the blood flow of 200 mL/min, dialysate flow of 200 mL/min, and saline flushes instead of anticoagulation. The cohort consisted of three subgroups: patients undergoing LTx with preoperative serum creatinine (sCr) < 2 mg/dL (Group 1: *n* = 22), patients undergoing LTx with preoperative sCr ≥ 2 mg/dL (Group 2: *n* = 73), and patients undergoing simultaneous liver-kidney transplantation (Group 3: *n* = 7). Among all the transplantations, 30% were retransplantations. The mean calculated MELD (Model for End-Stage Liver Disease) score was 39.2 in Group 2, with 67% of patients being hospitalized in the intensive care unit (Table 2). In patients from group 1, despite being less ill, severe intraoperative derangements developed, and they required urgent IOKRT, which was introduced significantly later than in group 2. In all subgroups, potassium levels <4 mmol/L were achieved postreperfusion, as well as a decrease in central venous pressure. Clots in the dialysis circuit were observed in 6.8% of cases. No other significant technical complication was observed. Postreperfusion syndrome occurred in 12.7% of patients [57]. Thirty-five patients in the cohort died in-hospital. Elevated mortality was likely due to the high illness severity in the cohort. On discharge, none of the patients required kidney replacement therapy. However, among the 35 patients who died during hospital stay, 25 were on dialysis until death (two in Group 1, 22 in Group 2, and one in Group 3). In this specific study, IOKRT was performed using a mobile Genius^®^ single-pass batch dialysis system *via* a dedicated dual-lumen central venous catheter, with no anticoagulation. The extracorporeal circuit was flushed with 100 mL of normal saline every 30–60 min. On the other hand, in our own protocol, the default blood and dialysate flow rates were set to 200 mL/min, and the dialysate potassium rate was set to 2 mmol/L, unless the nephrologist decided otherwise based on each patient’s clinical and laboratory status. A 90-L thermally insulated dialysis fluid container allowed us to perform 450 min of dialysis. If necessary, two machines were used. Hemodialysis was initiated by hemodialysis nurses but continued during surgery without their on-site involvement, under the supervision of the anesthesiologist.

**Table 2.** Characteristic of patients undergoing intraoperative kidney replacement therapy during liver transplantation in studies by Kościelska et al. [57] and Nadim et al. [59].

	Kościelska et al.	Nadim et al.
Number of patients	102	238
Blood flow, ml/min	200	200–300
Dialysate flow, ml/min	200	200–300
Anticoagulation	saline flushes	saline flushes
Baseline serum creatinine level (SCr), mg/dl	4	3 ± 1.7
Average MELD score	34.4 ± 12.1	37 ± 7.4
Average operative time, hours	6.25	9.4 ± 2.9
Number of patients -subgroups:		
Liver transplant alone - baseline SCr < 2 mg/dl	22	–
Liver transplant alone - baseline SCr > 2mg/dl	73	–
Liver transplant alone	–	155
Simultaneous liver-kidney transplantation	7	83
MELD - subgroups:		
Liver transplant alone - baseline SCr < 2 mg/dl	22.7 ± 10.5	–
Liver transplant alone - baseline SCr > 2mg/dl	39.2 ± 9.5	–
Liver transplant alone	–	39 ± 6.4
Simultaneous liver-kidney transplantation	21.1 ± 0.8	35 ± 7.8
Operative time, hours - subgroups		
Liver transplant alone - baseline SCr < 2 mg/dl	6.2	–
Liver transplant alone - baseline SCr > 2mg/dl	6	–
Liver transplant alone	–	7.9 ± 1.9
Simultaneous liver-kidney transplantation	9.5	11.8 ± 2.7

In the study by Nadim et al. [59], 155 CKRT procedures were performed as IOKRT procedures with blood flow of 200–300 mL/min and dialysate flow of 200–300 mL/min, using regular saline flushes instead of anticoagulation (Table 2). No significant complications were reported [59]. In a meta-analysis performed by Huang et al. [58], which included seven studies, the modalities used as IOKRT were: CVVHDF in 3, CVVHF in 2, and CVVHD in one, 426 IOKRT procedures were analyzed. Patients who received IOKRT had higher MELD scores and higher long-term mortality rates than those without IOKRT. However, short-term mortality did not differ between the groups [58].

IOKRT is a procedure that allows patients with kidney failure to receive liver transplantation, which is lifesaving. Considering conditions associated with liver failure, the use of SLED as a method for IOKRT appears to be the most appropriate choice. Additionally, no evidence has been found to support the superiority of CKRT over SLED [35].

#### SLED in heart failure

The hallmark of most patients with heart failure is fluid overload and congestion. Signs and symptoms of congestion characterize acute decompensated heart failure (ADHF). It is associated with an increased risk of multiple organ dysfunction, prolonged and recurrent hospitalization, and mortality [60]. The management is targeted at systemic decongestion, which is considered a treatment success [61]. Kidney dysfunction or worsening of kidney function appear to be an essential contributor to morbidity and mortality in patients with advanced HF [62,63] Cardiorenal syndrome is defined as the bidirectional relationship between the two organs: heart and kidneys, where the failure of one organ promotes pathological changes in the other [64,65] CRS is diagnosed in almost half of the patients with HF [64,66]. The management of these patients is a challenging task. In patients with CRS and other compelling conditions such as metabolic acidosis, electrolyte disturbances, volume overload, and uremia, KRT is considered [67]. However, the choice of extracorporeal techniques—intermittent, hybrid, or continuous—is mainly based on experience, rather than evidence, as data on the choice of therapy are limited. Several trials comparing different modalities were conducted in various critical care environments and provided inconclusive and conflicting evidence [39,68,69]. Zhang et al. [38], in a meta-analysis of 17 studies including critically ill patients with acute kidney injury, with 31% in a cardiac intensive care unit, reported that SLED as the form of the KRT was not associated with a different clinical course, renal recovery, or survival as compared with CVVHDF. However, this hybrid therapy could be beneficial, providing better hemodynamic stability, offering earlier and more profound rehabilitation, and reducing the need for healthcare resources. KRT in HF should be adapted to the clinical characteristics of the patients. Wojtaszek et al. [70] recently proposed an algorithm for selecting a KRT technique in HF patients.

#### SLED in sepsis

Chhallani [71] stated very elegantly that there is insufficient data available on the comparison of SLED and CKRT in sepsis-associated kidney injury. Mishra et al. [72] performed a pilot randomized controlled trial in septic patients. A study from India demonstrated the cost-effectiveness of SLED compared to CKRT. Taha et al. [73] published data from a single-center prospective study in sepsis-associated AKI of hemodynamically unstable patients in Sudan. They enrolled 15 patients in the SLED group and 13 in the CKRT group. They found that outcomes such as renal recovery, length of ICU stays, and dialysis recovery did not differ significantly between groups; however, the sample size was small. They stressed that SLED was cost-effective and covered by medical insurance; however, neither the government nor private medical insurance reimburses the cost of CKRT in Sudan. Most recently, Alam et al. [74] in a single-center, prospective, observational study assessed short-term outcomes in critically ill Indian patients with acute kidney injury (59.2% with sepsis) requiring KRT in a form of either IHD or SLED. In their study, 30-day dialysis-free survival and 3-day kidney recovery were similar. They concluded that SLED was a feasible, affordable, and cost-effective modality for hemodynamically unstable AKI patients, in resource-limited settings.

In critically ill patients with AKI, both modalities are safe and effective, as shown by meta-analysis and systematic reviews [17,38,40,75–78]. It was also advocated that the focus of ICUs be shifted toward implementing and optimizing SLED programs, particularly in countries/regions where CKRT is not readily available [74], to improve global care equity and outcomes for critically ill patients with AKI requiring KRT.

#### SLED and ECMO

The evidence of circuit configuration for ECMO is only emerging. Different combinations of kidney replacement therapy and ECMO circuits have been reported as KRT can be performed independently using a specific vascular access or directly integrated into the ECMO circuit [79,80]. This is an ‘integrated’ and a ‘parallel’ configuration of ECMO and KRT [78,79]. As reported by Sherwin et al. [81] parallel KRT had a significantly longer initiation time and shorter filter life, with higher rates local bleeding. CKRT could be also combined with ECCO_2_R (extracorporeal carbon dioxide removal), due to similar flow rates, making a comprehensive lung and renal support system possible [82].

In a study by Arnold et al. SLED and ECMO were used in 9 patients with ARDS associated with COVID-19 infection and concomitant acute kidney injury [83]. They used the Genius system, which was incorporated with ECMO and placed between sampling outlets of the oxygenators. The combination of ECMO-SLED devices was effective in reducing serum creatinine and urea levels, as well as in reducing the required ultrafiltration rate. The average lifespan of the filter was long, with a median of 18.3 h. In the recent review, Lau et al. [84] discussed increasing use of ECMO, in part due to the COVID-19 pandemic. However, they also underlined that currently used types of KRT are currently being used and outcomes with concurrent ECMO use across centers may be mixed. Izutani et al. [85] very recently reported use of ECMO together with KRT in patients with severe COVID-19, they also stressed that COVID-19 complicated with AKI had much worse prognosis, despite combined therapy ECMO with CKRT.

Osterman and Lumlertgul [86] presented the possibility of providing CKRT, SLED, and IHD *via* integration into the ECMO circuit or separately in an exquisite manner. They also emphasized that the advantages of the ECMO-SLED combination include reduced filter downtime, lower costs compared to CKRT, and lower volume and solute removal compared to IHD. In our experience, SLED was initiated in patients treated with ECMO using different vascular access methods. However, in patients with vascular access problems, SLED (access-inlet and return-outlet) was connected before the centrifugal blood pump (low-pressure part) of the ECMO circuit. Lumlertgul et al. [87] published long-term mortality and kidney outcomes in adult patients treated with veno-venous ECMO; however, data on the type of KRT modality were not provided.

## Limitations

Although SLED shows promise in terms of safety, efficacy, and sustainability, several limitations remain. High-quality randomized trials are limited, and their use is still highly dependent on local expertise, protocols, and equipment availability. The prolonged duration of treatment may pose logistical challenges, particularly in units with limited dialysis capacity, particularly when machine availability is constrained. Additionally, environmental benefits vary by system, and in some configurations, water consumption may exceed that of CKRT. The absence of consistent guideline recommendations further limits its broader adoption. These factors underscore the need for more standardized protocols and robust clinical data. Although there is considerable data to support SLED treatment for critically ill adult patients with AKI, there are fewer pediatric data, even so, as stressed by Sethi et al. [88] currently available studies support the use of SLED for the pediatric population, particularly in resource-limited settings.

## Summary

The introduction of sustained low-efficiency dialysis has been considered a breakthrough in kidney replacement therapy, not only in acute kidney injury but also in other settings. According to Raina et al. [89], SLED was used in 25% of centers in developing countries, compared to 20% in developed countries, as reported in a recent survey conducted in 60 US centers and 48 in the Indian subcontinent and Latin America. The largest advantage of SLED is simply the ease of availability with minimized cost. Moreover, especially in nonacademic medical centers, to bring in more than one type of platform for CKRT is probably an invitation for medical errors. In most cases, SLED technology also represent a ˝hybrid˝ modality in terms of work assignment; whereas classic CKRT run exclusively by ICU nurses (with supervision by nephrologist being country and center-dependent), running SLED program typically incorporate nursing help from the inpatient dialysis unit (either in setting up the platform or helping to retain skills in low-volume centers). SLED provides similar outcomes in terms of urea removal, comparable to those obtained by CKRT, in shorter treatment time and with reduced overall resource utilization. Due to its hemodynamic tolerability and feasibility and being less costly than CKRT, SLED is equally safe and effective in treating AKI among severely ill patients [12,31,33,34,90].

In addition, Sethi et al. [88] concluded in their paper that during the COVID-19 pandemic, the shortage of CKRT equipment led to increasing usage of SLED as an alternative treatment for acute kidney injury. Table 3 present clinical comparison of SLED vs CRRT as a guidance for therapy selection.

**Table 3.** A summary table comparing clinical aspects of SLED and CRRT to guide therapy selection.

Feature/Clinical Scenario	SLED (Sustained Low-Efficiency Dialysis)	CRRT (Continuous Renal Replacement Therapy)
Hemodynamic Stability	Very good, comparable to CRRT	Gold standard for extremely unstable patients
Small-Molecule Clearance (e.g., urea, creatinine)	High efficacy due to dominant diffusion; rapid azotemia control	Moderate but constant; slower metabolic control
Middle-Molecule Clearance (e.g., cytokines)	Moderate (dependent on high-flux membrane)	Potentially higher (especially in CVVH/CVVHDF with high convection)
Anticoagulation	Often not required (saline flushes are feasible)	Usually required (heparin or citrate); citrate is standard but requires monitoring
Patient Mobility/Access to Procedures	Allows for ‘therapy-free’ windows during the day for rehabilitation, diagnostics, and procedures	24–72 hour therapy, significantly limits patient mobility
Staffing/Logistics	Less burdensome for ICU staff (often managed by dialysis nurses), shorter duration	Requires constant ICU staff supervision, more labor-intensive
Cost	Significantly lower (fewer consumables, no expensive pre-made fluids)	High (cost of filters, pre-made fluids, citrate anticoagulation)
Preferred Indication	Hemodynamic instability, need for patient mobilization, high bleeding risk, resource-limited settings	Refractory shock, severe sepsis with high catabolism, acute brain injury with edema risk

## Conclusions

SLED could be a viable option as a kidney support during liver transplantation, for acute decompensated heart failure patients, and for sepsis as well as some other setting as an alternative to CKRT. It will be more beneficial for both patients and the environment, promoting environmental protection. Such an approach is needed in the context of climate change and is crucial for sustainable development and kidney care. Besides being ‘green’, SLED is less labor-intensive and less expensive relative to CKRT and hence can be a practical alternative in settings with limited resources and expertise. Thus, we express our optimism in holding on to this hope until sufficient data is available. Therefore, in the context of green nephrology SLED, the old method could be a new solution for environmental nephrology.

Author contribution: conception and design: Aleksandra Rymarz and Jolanta Malyszko, analysis and interpretation of the data: Aleksandra Rymarz, Jolanta Malyszko, Pawel Zebrowski, Malgorzata Kepska-Dzilinska, Zuzanna Jakubowska, Filip Wantoch-Rekowski, drafting of the paper: Aleksandra Rymarz, Jolanta Malyszko,Pawel Zebrowski, Malgorzata Kepska-Dzilinska, Zuzanna Jakubowska, Filip Wantoch-Rekowski, Ewa Wojtaszek revising it critically for intellectual content; Aleksandra Rymarz, Jolanta Malyszko, Pawel Zebrowski and the final approval of the version to be published: Aleksandra Rymarz, Jolanta Malyszko,Pawel Zebrowski, Malgorzata Kepska-Dzilinska, Zuzanna Jakubowska, Filip Wantoch-Rekowski, Ewa Wojtaszek.

## Data Availability

No new data were generated
